# A Novel SNP Associated with Nighttime Pulse Pressure in Young-Onset Hypertension Patients Could Be a Genetic Prognostic Factor for Cardiovascular Events in a General Cohort in Taiwan

**DOI:** 10.1371/journal.pone.0097919

**Published:** 2014-06-03

**Authors:** Hsin-Bang Leu, Chia-Min Chung, Shing-Jong Lin, Tse-Min Lu, Hsin-Chou Yang, Hung-Yun Ho, Chih-Tai Ting, Tsung-Hsien Lin, Sheng-Hsiung Sheu, Wei-Chuan Tsai, Jyh-Hong Chen, Wei-Hsian Yin, Ting-Yu Chiu, Chin-Iuan Chen, Wen-Harn Pan, Jaw-Wen Chen

**Affiliations:** 1 Institute of Clinical Medicine and Cardiovascular Research Center, National Yang-Ming University, Taipei, Taiwan; 2 Heath Care and Management Center, Taipei Veterans General Hospital, Taipei, Taiwan; 3 Divison of Cardiology, Department of Medicine, Taipei Veterans General Hospital, Taipei, Taiwan; 4 Institute of Pharmacology, National Yang-Ming University, Taipei, Taiwan; 5 Institute of Biomedical Sciences, Academia Sinica, Taipei, Taiwan; 6 Institute of Statistical Science, Academia Sinica, Taipei, Taiwan; 7 Taichung Veterans General Hospital, Taichung, Taiwan; 8 Kaohsiung Medical University Chung-Ho Memorial Hospital, Kaohsiung, Taiwan; 9 National Cheng Kung University Hospital, Tainan, Taiwan; 10 Cheng Hsin Rehabilitation Medical Center, Taipei, Taiwan; 11 Min Sheng General Hospital, Taoyuan, Taiwan; Shenzhen Institutes of Advanced Technology, China

## Abstract

**Background:**

Pulse pressure (PP) is a risk factor for cardiovascular disease. It has been reported that ambulatory blood pressure (BP) and nighttime BP parameters are heritable traits. However, the genetic association of pulse pressure and its clinical impact remain undetermined.

**Method and Results:**

We conducted a genome-wide association study of PP using ambulatory BP monitoring in young-onset hypertensive patients and found a significant association between nighttime PP and SNP rs897876 (p = 0.009) at chromosome 2p14, which contains the predicted gene FLJ16124. Young-onset hypertension patients carrying TT genotypes at rs897876 had higher nighttime PP than those with CT and CC genotypes (TT, 41.6±7.3 mm Hg; CT, 39.1±6.0 mm Hg; CC, 38.9±6.3 mm Hg; p<0.05,). The T risk allele resulted in a cumulative increase in nighttime PP (β = 1.036 mm Hg, se. = 0.298, p<0.001 per T allele). An independent community-based cohort containing 3325 Taiwanese individuals (mean age, 50.2 years) was studied to investigate the genetic impact of rs897876 polymorphisms in determining future cardiovascular events. After an average 7.79±0.28 years of follow-up, the TT genotype of rs897876 was independently associated with an increased risk (in a recessive model) of coronary artery disease (HR, 2.20; 95% CI, 1.20–4.03; p = 0.01) and total cardiovascular events (HR, 1.99; 95% CI, 1.29–3.06; p = 0.002), suggesting that the TT genotype of rs897876C, which is associated with nighttime pulse pressure in young-onset hypertension patients, could be a genetic prognostic factor of cardiovascular events in the general cohort.

**Conclusion:**

The TT genotype of rs897876C at 2p14 identified in young-onset hypertensive had higher nighttime PP and could be a genetic prognostic factor of cardiovascular events in the general cohort in Taiwan.

## Background

Hypertension is a leading cause of death, especially in highly industrialized regions, and is considered to be the major risk factor for cardiovascular disease [1]. Recent large-scale genome-wide association studies (GWAS) have reported more than 20 novel loci for systolic and diastolic blood pressure (SBP and DBP) where alleles have effect sizes of up to 0.5–1 mm Hg [2]–[4]. Although the genetic impact of determining BP value is quite small, increments in BP still have important effects on cardiovascular morbidity and mortality at the population level [5].

Pulse pressure (PP), the difference between systolic blood pressure and diastolic blood pressure, represents the hemodynamic load on the vasculature and indirectly measures central aortic stiffness. It has been reported that higher PP is associated with left ventricle hypertrophy [6] and the increased intimal thickness of the carotid artery [7], which represent early target organ damage in cardiovascular diseases. Furthermore, increased PP has been reported to be associated with a higher risk of developing advanced cardiovascular events, such as myocardial infarction [8], stroke [9], congestive heart failure [10] and cardiovascular death [11], suggesting that increased PP may cause cardiovascular organ damage and could be seen as an important predictor leading to poor outcomes. Recently, GWAS have also focused on this specific phenotype of blood pressure for hypertension research, and several novel loci related to PP were found [12]. Interestingly, the effects of these PP-related loci on systolic BP (SBP) and diastolic BP (DBP) significantly differ from the effects of loci found in previous GWAS of either SBP or DBP, suggesting the possibility of novel genetic mechanisms underlying blood pressure variation. However, the exact mechanisms underlying the modulation of PP remain undetermined.

Ambulatory blood pressure (ABP) monitoring is a validated and accurate method to evaluate blood pressure during a 24-hour period. The correlation between blood pressure (BP) level and target organ damage, cardiovascular disease (CVD) risk, and long-term prognosis is greater via ABP monitoring than official BP measurements. Additionally, PP heritability is higher (0.47–0.57) when using ABP measurements rather than single-point measurements. This indicates that higher heritability is associated with an increased number of BP measurements; therefore, ABP monitoring is more appropriate for the study of the genetics of essential hypertension [13]. Therefore, we conducted a genetic association study of the PP, as measured by ABP monitoring of hypertensive patients. To increase the genetic influence and homogeneity of the study trait, young-onset hypertension subjects with a strong genetic component to their hypertension were selected for investigation in this study [14]. In addition, to further investigate the clinical impact of the genetic association of ABP monitoring, a subsequent cohort study was conducted to investigate the genetic impact of PP-related loci in determining future cardiovascular outcomes.

## Methods

### Study Subjects

#### (1) Genetic association of ambulatory pulse pressure in young-onset hypertension

A two-step genetic association analysis of young-onset hypertension was performed to analyze the genetic association of ambulatory PP in young-onset hypertension. In the first step, a GWAS was conducted with young-onset hypertensive patients as the discovery group (n = 382) to find significant SNP markers. In the second step, a replication study was conducted to test whether SNP markers filtered in first stage were significantly associated with PP in the replication samples (n = 559). The diagnostic criteria for young-onset hypertension have been published previously [15] and were defined as systolic blood pressure (SBP) higher than 140 mm Hg and diastolic blood pressure (DBP) higher than 90 mm Hg over a 2-month period or the use one type of antihypertensive medication. A diagnosis of young-onset hypertension occurs between 20 and 51 years of age. Secondary causes of hypertension, such as chronic renal disease, renal arterial stenosis, primary aldosteronism, coarctation of the aorta, thyroid disorders, Cushing syndrome or pheochromocytoma were excluded through extensive clinical examinations and investigations including blood chemistry and endocrinology tests. Furthermore, individuals with a diagnosis of diabetes mellitus (fasting glucose>126 mg/dl) or marked obesity (BMI>35 Kg/m2) were also excluded from this study. All enrolled hypertensive patients received 24-h ambulatory blood pressure monitoring (ABPM). The ABPM was attached to the upper left arm. The BP measurements were based on Korotkoff sounds during stepwise deflations (3.0±1.0 mm Hg/step) of the cuff. Both BP and heart rate measurements were obtained at 30-min intervals. Noninvasive ABPM was performed on a weekday with 1 of 3 automatic devices that recorded BP and pulse rate every 30 minutes for 24 hours. For each recording, the study patients were recommended to go to bed at 23∶00 at night and wake up at 07∶00 in the morning. Ambulatory BP values were edited for artifacts using preselected criteria as described in previous studies [16], and the daytime (7∶00 AM to 11∶00 PM) values were averaged, as were the nighttime values (11∶00 PM to 7∶00 AM). The patients were also asked to record their real sleeping time if it differed from the specified range. The BP measurements during sleep were then used to calculate the nighttime BP, and the remaining BP recordings were used to calculate the daytime BP. The associations of genotype with distinct quantitative traits, including daytime and nighttime PP, were analyzed separately. The accuracy of these devices was validated in our previous study [17]. This study protocol was approved by the Human Investigation Committee of the Institute of Biomedical Sciences, Academia Sinica and each participating hospital: Taipei Veterans General Hospital, Taichung Veterans General Hospital, Kaohsiung Medical University Chung-Ho Memorial Hospital, National Cheng Kung University Hospital, Cheng-Hsin Rehabilitation Medical Center, and Min-Sheng General Hospital. Written informed consent was obtained from each subject.

#### (2) Impact of genetics in determining future cardiovascular event in an independent cohort

To determine the clinical implications of a genetic association with pulse pressure found in young-onset hypertension, CardioVascular Disease risk FACtors Two-township Study (CVDFACTS), a cohort study (n = 3325), was selected for investigating the association of future cardiovascular events and the genetic risk variants. The CVDFACTS cohort is a community-based follow-up study begun in 1989 to investigate the cardiovascular disease occurrence and risk factors in Taiwan [18]–[20]. Briefly, five villages with more than 1000 people and a population density greater than 200 people per square kilometer were randomly selected from Chu-Dong (northwest Taiwan) and Pu-Tzu (southwest Taiwan). Information about participants’ lifestyle, risk factors, history of cardiovascular disease, and urine and blood chemistry were collected. Repeated examinations were conducted in 1989–1990, 1990–1993, 1994–1997, 1997–1999, and 2000–2002. Cardiovascular events, including ischemic stroke, fatal or non-fatal myocardial infarction and cardiovascular death were identified from a review of self-reported disease histories, death certificates, and insurance claim records of the National Health Insurance (NHI) database dated until the end of 2002. Subjects without a history of stoke and coronary artery disease were enrolled and tracked in the NHI database after 1995, the year the NHI database was generated. The NHI included 99.5% of our subjects. The cardiovascular events were identified from NHI database records using codes 430 to 438 for ischemic stroke and 410–414 for coronary artery disease (CAD) from the International Classification of Diseases, 9th Revision, Clinical Modification (ICD-9-CM). Cardiovascular death was identified from death certificates. We used the National Death Registry database, which obtains information from certified death certificates and codes death according to the International Classification of Disease, Ninth Revision. The sensitivity and specificity for event identification were 100% and 95%, respectively [18]–[20].

### Power Calculations

We calculated the power of our two-stage genetic association study using CaTS software [21]. Given an additive-effect disease model with a prevalence of 15% for young-onset hypertension [22], a genetic relative risk of 2, and a disease allele frequency of 0.2–0.3, the power of our two-stage analysis was 0.80–0.85. The power was reduced to 0.05–0.09 if the genetic relative risk was reduced to 1.5. Similar power calculations have been performed in our previous work [23].

**Figure 2:**
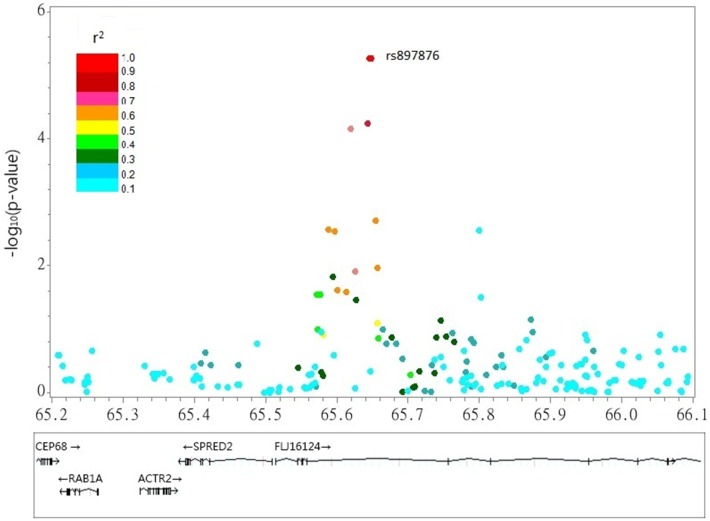
Plot of rs897876 located in the 2p14 locus associated with nighttime PP.

### Genotyping Methods

Genomic DNA was extracted from peripheral blood samples of hypertensive subjects using the Puregene DNA isolation kit (Gentra Systems, Minneapolis, MN, USA) for the young-onset hypertension genetic study and using the phenol/chloroform method for CVDFACTS. For the GAWS, genotyping experiments were performed with leukocyte DNA using the Illumina Infinium II HumanHap550 SNP chips (Illumina, San Diego, CA, USA), which included 560,186 tagging SNPs selected from phase I and II of the HapMap Project. Genotyping assays were performed by deCODE genetics (Reykjavik, Iceland) for 382 young-onset hypertensive subjects in the first stage of the study. We followed the WTCCC criteria for quality control; in brief, individuals were excluded if more than 3% of the genotype data were missing. SNPs were excluded if they showed violation of the Hardy-Weinberg equilibrium (p<1×10^−7^), call rates <95%, or minor allele frequency <1%. Genotyping for the verification study and subsequent genotyping of subjects in the CVDFACTS study were conducted using the Sequenom MassARRAY System (San Diego, CA, USA) by the Academia Sinica National Genotyping Center (Taipei, Taiwan).

### Statistical Analysis

The discovery GWAS with Illumina Infinium II HumanHap550 SNP chips was usedas a general linear model to investigate the association of ambulatory BP parameters with genotype data, making adjustments for gender, age, BMI and medications for BP control. Because all of the patients with young-onset hypertension received 24-hour ambulatory BP recording, the association of genotypes with the quantitative traits (daytime and nighttime PP) were analyzed separately. To estimate the effect of genetics in determining ambulatory BP, a stepwise linear multiple regression model was used. The genome-wide significance threshold for the p-value was 5×10^−8,^ which included multiple testing correction. The EIGENSTRAT utility of the EIGENSOFT package version 2.0 [24] was used to quantify and correct for population sub-structure and adjust for population stratification in association analyses. Top ten Eigen vectors of the covariance matrix between the initial stage and the second stage were evaluated. All association analyses were performed using the PLINK software program [25] and SAS software version 9.2 (Cary, North Carolina, USA). For the community cohort study, the significance of between-group differences in means was assessed using Student’s t-test or ANOVA as appropriate, and the significance of differences between two proportions was tested with the chi-square test. Data on outcomes were censored either at the time of a cardiovascular event development or at the end of follow-up. A Kaplan-Meier curve and log-rank test for event-free survival were constructed for genetic variants. All p-values were two-sided and derived from likelihood-ratio statistics from Cox proportional-hazards regression models after adjusting for known risk factors, including age, gender, smoking, lipid profile, history of hypertension, and diabetes.

## Results

**Figure 1:**
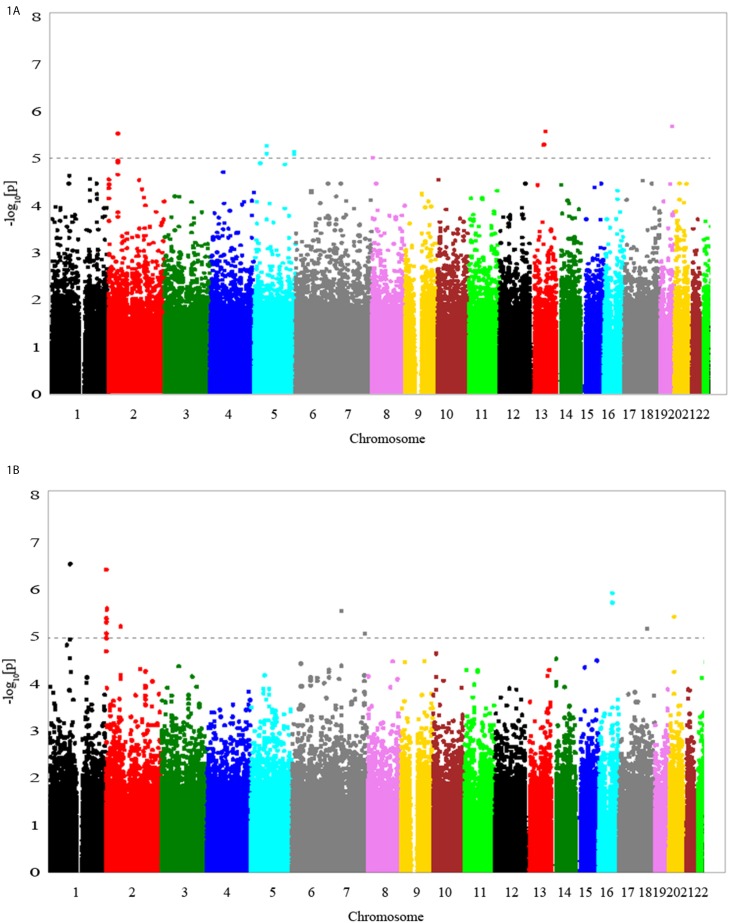
Signal-intensity plots showing the genome-wide associations of single-nucleotide polymorphisms (SNPs) with daytime PP (1A) and nighttime PP (1B) in young-onset hypertensives.

**Table 1** shows the baseline characteristics of young-onset hypertension patients in the discovery GWAS and the subsequent replication study. The p-values from the discovery GWAS for association tests of daytime PP and nighttime PP are shown in Figure 1. The top scoring SNPs for association with ambulatory PP parameters are shown in **Table 2**. The SNP with the lowest p-value (2.72×10^−7^) for the trait of nighttime PP was rs6696698, located on chromosome 1 (**Figure 1A**). The EIGENSTRAT utility of the EIGENSOFT package version 2.0 [24] was used to quantify and correct for population sub-structure and adjust for population stratification in association analyses. There was no evidence of population stratification in the hypertensive subjects (Table S1). Multidimensional scaling analysis using PLINK also showed similar results (Fig. S1). In the GWAS analysis no SNP exceeded the GWAS significance threshold for either of the ABP-monitoring phenotypes (daytime or nighttime PP). For each trait, considering that some SNP markers in the same area were in moderate linkage disequilibrium, all SNP markers achieving a –log p value more than 5 with a minor allele frequency more than 5% were selected in the replication study **(** **Table 2** **)**. Only one SNP marker, rs897876, which is in the predicted gene FLJ12164 at 2p14, was significantly associated with nighttime PP (p = 0.009) in the replication study (**Table 2** **, Figure2**). **Table 3** showed the baseline characteristics of these young-onset hypertension subjects according to genotypes of rs897876. Subjects with the TT genotype at rs897876 had higher nighttime PP values than those with the CT or CC genotype (TT, 41.6±7.3 mm Hg; CT, 39.1±6. 3 mm Hg; CC, 38.9±6.3 mm Hg; p<0.05). After regression analysis adjusting for age, gender, BMI, and hypertension treatment regimens, the T allele of rs897876 was additively associated with increased nighttime PP (β = 1.036 mm Hg, se. = 0.298, p<0.001 per T allele).

**Table 1:** Baseline characteristics of young-onset hypertension subjects.

	Discovery sample (n = 382)	Replication sample (n = 559)
Male, n(%)	260 (68)	387 (69.2)
Age, years	38.4±8.7	42.9±5.2
BMI, kg/m^2^	26.2±3.8	26.7±3.2
Waist circumference, cm	88.0±10.1	89.3±9.2
BP, mm Hg		
Systolic	125.2±15.0	126.9±14.3
Diastolic	83.9±11.9	85.6±11.7
Metabolic profiles		
Total cholesterol, mg/dl	195.3±37.1	196.7±35.2
HDL-C, mg/dl	46.5±12.1	44.8±12.2
Triglycerides, mg/dl	149.8±96.9	176.2±118.4
Glucose, mg/dl	96.7±8.9	96.0±9.4
Uric Acid, mg/dl	6.6±1.8	6.7±1.7
24-hours ambulatory BP recording		
Awake		
SBP, mm Hg	127.0±13.3	125.6±12.3
DBP, mm Hg	85.3±10.3	84.8±9.7
PP, mm Hg	41.7±7.0	40.8±6.3
Sleep		
SBP, mm Hg	114.9±13.2	113.6±12.4
DBP, mm Hg	74.6±10.6	74.9±9.5
PP, mm Hg	40.3±6.6	38.7±6.3
Antihypertensive medications		
ACE inhibitor/ARB, n (%)	121 (31.7)	219 (38.1)
β-blockade, n (%)	148 (38.7)	267 (47.8)
Calcium channel blockade, n (%)	120 (31.4)	232 (41.5)
Alpha blockade, n (%)	2 (0.5)	18 (3.2)
Diuretics, n (%)	39 (10.2)	58 (10.4)

Data are n (%) or mean ± SD; BMI, body mass index; HDL-C, high-density lipoprotein cholesterol; ACE, angiotensin-converting enzyme; ARB, Angiotensin II receptor blockers.

**Table 2:** Genetic associations with ambulatory BP parameters.

Chr	SNP (Gene)	Trait	*P* in the discoveryGWAS sample	*P* in the replicationsample	*P* in the combinedsample	MAF
5	rs6556114	Daytime PP	6.09 e^−6^	0.869	1.00 e^−3^	0.055
5	rs897669 (**DHX29**)	Daytime PP	3.33 e^−6^	0.33	3.06 e^−5^	0.067
8	rs2527083	Daytime PP	2.67 e^−5^	0.153	1.35 e^−2^	0.417
13	rs505299	Daytime PP	3.88 e^−6^	0.99	2.64 e^−4^	0.105
13	rs9564376 (**PCDH9**)	Daytime PP	5.03 e^−6^	0.221	1.10 e^−4^	0.085
1	rs6696698	Nighttime PP	2.72 e^−7^	0.898	1.48 e^−3^	0.187
2	rs17039365 (**MYT 1L**)	Nighttime PP	4.33 e^−6^	0.878	4.59 e^−4^	0.134
2	rs17338512 (**MYT 1L**)	Nighttime PP	5.34 e^−6^	0.835	1.75 e^−3^	0.132
2	rs7596980 (**MYT 1L**)	Nighttime PP	4.33 e^−6^	0.891	7.52e^−4^	0.135
2	rs897876 (**FLJ16124**)	Nighttime PP	5.58 e^−6^	0.009*	1.88 e^−7^	0.406
7	rs1019102	Nighttime PP	2.45 e^−6^	0.972	1.69 e^−2^	0.146
7	rs17513926 (**CNTNAP2**)	Nighttime PP	7.49 e^−6^	0.612	1.62 e^−4^	0.229

**Table 3:** Baseline characteristics of rs897876 in young-onset hypertension subjects.

	CC	CT	TT
	n = 332	N = 453	N = 156
Male, n(%)	226 (68)	301 (66.4)	102 (65.3)
Age, years	40.7±7.27	41.4±7.1	40.7±7.5
BMI, kg/m^2^	26.2±3.5	26.7±3.3	26.4±3.7
Waist circumference, cm	87.9±9.4	89.5±9.7	88.5±10.0
BP, mm Hg			
Systolic	125.9±15.0	126.3±14.4	127.4±15
Diastolic	84.9±11.9	85.1±11.5	84.6±12.6
Metabolic profiles			
Total Cholesterol, mg/dl	194.8±37.0	196±35.6	200.2±35.8
HDL-C, mg/dl	45.2±12.0	44.9±12.2	47.5±11.4
Triglycerides, mg/dl	161.4±107	171.2±116.2	157.4±109.5
Glucose, mg/dl	97.1±9.0	98.7±9.6	97.7±8.8
Uric acid, mg/dl	6.7±1.8	6.6±1.8	6.6±1.5
24 hours ambulatory BP recording			
Daytime			
SBP, mm Hg	125.8±12.4	126.4±12.2	127.1±14.5
DBP, mm Hg	84.8±9,8	85.5±9.6	85.1±11.6
PP, mm Hg	41.0±6.1	41.0±6.8	42.0±7.1
Nighttime			
Systolic BP, mm Hg	113.9±12.8	113.8±12.2	116.5±14.5
Diastolic BP, mm Hg	75.0±10.0	74.7±9.7	75.1±11.1
PP, mm Hg	38.9±6.3	39.1±6.3	41.6±7.3*

Data are n (%) or mean ± SD; BMI, body mass index; HDL-C, high-density lipoprotein-cholesterol;

*P<0.05 TT *vs.* CC and CT genotypes.

**Figure 3:**
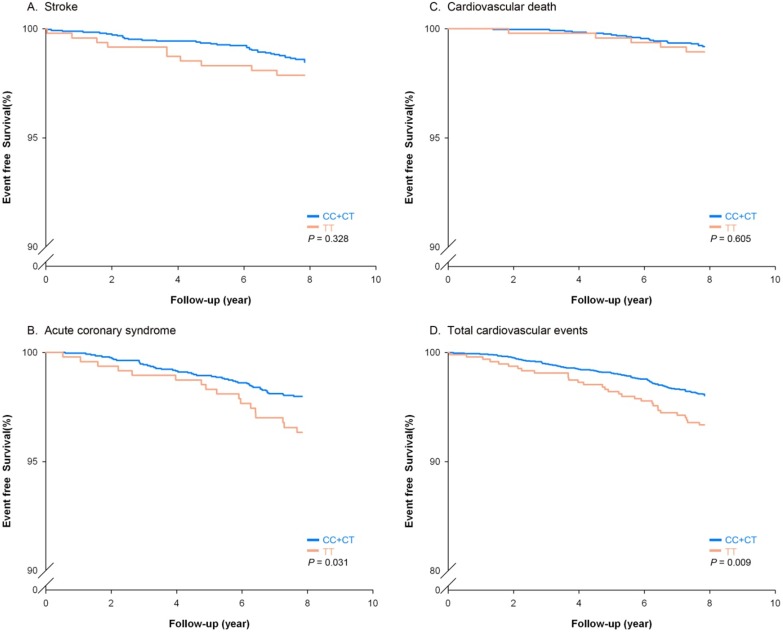
Kaplan-Meier estimates of survival-free cardiovascular events including stroke (A), acute coronary syndrome (B), cardiovascular death (C) and total cardiovascular events (D) according to rs897876 genotypes in a cohort study. The event-free survival rates for acute coronary syndrome and total cardiovascular events were significantly different in TT *vs*. CC+CT genotypes (log-rank test, p = 0.0031 and p = 0.009, respectively).

To determine the clinical significance of the nighttime PP-associated SNP found among young-onset hypertensive, genotyping for rs897876 was performed in a prospective cohort study, the CVDFACTS study, to evaluate the association of future cardiovascular events with rs897876. The genotype call rate was above 99.4%. The CVDFACTS study enrolled a total of 3325 subjects, 1513 males and 1812 females, with a mean age of 50.2±12.3. The CVDFACTS cohort used in this study consisted of a total of 389 hypertensive patients, 204 (52%) of whom were young-onset hypertensive patients. The young onset hypertensive subjects have higher diastolic blood pressure and higher BMI. Subject with hypertension diagnosed after age 50 have higher fasting glucose, and higher triglyceride values (Table S2). After an average of 7.8 years of follow-up, 68 ischemic stroke events, 99 acute coronary syndrome events, 34 cardiovascular-related deaths and 190 total events were identified (**Table 4**). In this independent cohort, the T allele of rs897876 was significantly associated with CAD and total cardiovascular (CV) events (under a recessive model for the variant allele, log-rank p for CAD = 0.031 and for total CV events = 0.009) (**Table 5**). **Figure 3** shows the effect of rs897876 polymorphisms on clinical outcomes in the cohort participants. The TT genotype of rs897876 was associated significantly with a higher risk of developing CAD and total cardiovascular events. After adjusting for comorbidity, including history of diabetes mellitus, smoking habit, gender, hypertension, waist circumference, total cholesterol, BMI, and age, the TT genotype of rs897876 still independently associated with CAD (hazard ratio, 2.20; 95% CI, 1.20–4.03; p = 0.01) and total CV events (hazard ratio, 1.99; 95% CI, 1.29–3.06; p = 0.002) (**Table 6**), indicating that the TT genotype of rs897876C genotypes, which was identified based on the ambulatory night PP values of young-onset hypertension patients, was associated with a higher risk of future cardiovascular events.

**Table 4:** Baseline characteristics of subjects with future cardiovascular events in community-based cohort study.

	Total subjects	Subjects developing ischemic stroke	Subjects developing ACS	Subjects developing CV death	Subjects developing total events
	N = 3325	N = 68	N = 99	N = 34	N = 190
Age, years	50.2±12.3	59.0±8.8	59.9±9.7	65.5±7.8	60.3±9.4
Male, n (%)	1513 (45.5)	37 (54.4)	57 (57.6)	22 (64.7)	109 (57.4)
BMI, kg/m^2^	24.3±3.3	25.2±4.0	25.2±3.7	23.0±3.7	24.8±3.9
Diabetes, n (%)	90 (2.7)	7 (10.3)	10 (10.1)	3 (8.8)	17 (8.9)
Smoking, n (%)	605 (18.2)	18 (26.5)	33 (33.3)	12 (35.3)	58 (30.5)
Waist circumference, cm	81.0±9.4	84.5±11.0	86.0±9.8	81.4±10.7	84.5±10.4
SBP, mm Hg	120.1±18.0	132.3±20.8	131.8±20.3	132.6±23.7	131.7±21.1
DBP, mm Hg	73.3±11.5	79.8±9.7	78.0±12.0	75.9±9.1	78.3±11.0
Glucose, mg/dl	101.2±25.5	109.4±29.5	111.5±44.0	108.1±42.6	109.3±37.0
Triglycerides, mg/Dl	110.5±81.3	129.7±79.7	144.8±85.3	106.3±45.5	133.6±80.3
Cholesterol, mg/dL	198.0±44.2	205.7±46.9	218.3±43.1	193.6±37.1	209.7±45.0
HDL-C, mg/dL	43.1±14.7	40.5±10.7	39.4±11.8	43.2±11.4	40.3±11.4
LDL-C, mg/dL	128.8±42.6	126.5±41.1	135.1±38.2	120.4±27.5	128.9±38.5

Data are n (%) or mean ± SD; BMI, body mass index; HDL-C, high-density lipoprotein-cholesterol; LDL-C, low-density lipoprotein-cholesterol; ACS, acute coronary syndrome (unstable angina, myocardial infarction).

**Table 5:** Log-rank P models of the effect of the rs897876 genotype on cardiovascular events.

	Models		
	Additive	Dominant	Recessive
Stroke	0.112	0.186	0.328
CAD	0.078	0.051	**0.031**
CV death	0.801	0.612	0.605
Total CV events	0.091	0.574	**0.009**

CAD indicates coronary artery disease; CV death, cardiovascular death.

Total CV events includes unstable angina, myocardial infarction and stable CAD.

**Table 6:** Cardiovascular events and risks according to the rs897876 genotypes in cohort study.

	No. of subjects (%)	No. of event (%)	HR (95%CI)*	*P^e^*
**Stroke**				
CC	1077 (37)	21 (1.9)	reference	
CT	1373 (47)	16 (1.1)	0.62 (0.32–1.22)	0.17
TT	473 (16)	10 (2.1)	1.1 (0.05–2.4)	0.82
CC+CT	2450 (84)	37 (1.5)	reference	
TT	473 (16)	10 (2.1)	1.35 (0.65–2.82)	0.42
**CAD**				
CC	1077 (37)	19 (1.8)	reference	
CT	1373 (47)	30 (2.2)	1.93 (0.95–3.95)	0.71
TT	473 (16)	17 (3.6)	3.37 (1.52–7.47)	0.003
CC+CT	2450 (84)	49 (2)	reference	
TT	473 (16)	17 (3.6)	2.20 (1.20–4.03)	0.01
**CV death**				
CC	1077 (37)	10 (0.9)	reference	
CT	1373 (47)	10 (0.7)	0.74 (0.21–1.93)	0.54
TT	473 (16)	5 (1.1)	1.66 (0.55–4.99)	0.37
CC+CT	2450 (84)	20 (0.8)	reference	
TT	473 (16)	5 (1.1)	1.93 (0.70–5.29)	0.203
**Total CV events**				
CC	1077 (37)	44 (4.1)	reference	
CT	1373 (47)	52 (3.7)	1.06 (0.69–1.65)	0.78
TT	473 (16)	31 (6.6)	2.05 (1.25–3.36)	0.004
CC+CT	2450 (84)	96 (3.9)	reference	
TT	473 (16)	31 (6.6)	1.99 (1.29–3.06)	0.002

*HRs (95%CIs) and their corresponding *P* value were calculated using Cox proportional hazard models, adjusting for age, sex, smoking habit, blood pressure, BMI, lipid profiles and history of diabetes.

## Discussion

In our present study, we first identified rs897876 at 2p14, which is highly associated with nighttime pulse pressure in young-onset hypertension. Subjects who carried the TT genotype at rs897876 had higher nighttime PP, indicating that the T allele of rs898786 is an independent predictor associated with higher nighttime PP (β = 1.036 mm Hg, se. = 0.298, p<0.001 per T allele). Furthermore, the T allele of rs897876 was associated with an increased risk of developing future CAD and total cardiovascular events in an independent cohort. Consequently, the T allele of rs897876 could be a genetic prognostic factor for long-term outcomes in general cohort that include hypertensive patients and could be seen as a genetic marker for advanced cardiovascular events. Furthermore, 2p14 is a locus of interest for further investigation.

Recent large-scale GWAS have reported more than 20 novel loci for SBP and DBP where alleles have effect sizes of up to 0.5–1 mm Hg [2]–[4]. However, the GWAS of PP that used single-point BP values found that PP-associated loci differ from loci associated with SBP and DBP. Furthermore, the effects of PP-associated loci are distinct from the effects of SBP- and DBP-associated loci [12], suggesting that distinct mechanisms may underlie blood pressure variation. In addition, none of the genes in the previously identified PP-associated regions are strong candidates for blood pressure determination, and the clinical impacts of these loci remain undetermined. Our current study first identified the locus for increased nighttime PP using 24-hour ABP monitoring. This SNP marker could be a genetic prognostic factor for long-term outcomes in community-based cohorts, including the hypertensive subjects in Taiwan, suggesting a possible use for this marker in clinical practice.

It is interesting that different gene sets seem to contribute to the regulations of daytime and nighttime ambulatory BP. This finding is in accordance with previous findings that the gene sets that regulate daytime and nighttime blood pressure overlap, but there is a genetic component that is specific to the nighttime BP control [26], [27]. A previous study investigating the heritability of BP parameters demonstrated that PP had the highest heritability [28], suggesting that PP might be the most susceptible target influenced by genetic components. Furthermore, clinical observations also demonstrated clearly that PP correlated with target organ damage [8]–[11], suggesting that searching for PP genetic markers may have clinical value for identifying patients at risk. Although there is limited information about this SNP marker (rs897876), which is in a predicted gene located on chromosome 2p14, this SNP is near genes known to be related to the cardiovascular system. Recently, Ullrich et al. showed that *SPRED2* (65M) is a negative regulator of the hypothalamic-pituitary-adrenal axis and contributes to the modulation of hyperaldosteronism and homeostatic imbalances [29]. In addition, *RAB1A* (65M) has been reported to be associated with cardiomyopathy [30]. To look for genes involved in BP control, the HERITAGE family study used linkage scans to identify several loci, including 2p14, as possible candidates in modulating BP control [31]. Using meta-analysis based on genome-wide linkage studies, Rice et al reported that 2p14-p13.1 (64–78 cM) had a maximal LOD score, providing compelling evidence of its involvement in BP control [32]. Although there is little information about the causal genes in this region and little knowledge of how it modulates BP control., several genes near this region, including adducin (ADD2, 70 cM), G-protein–coupled receptor (GPR723, 68M), and transforming growth factor-α(TGFA, 70M), are associated with hypertension [31], [32]. Our study demonstrated that ambulatory nighttime PP has significant a genetic association and our results narrowed the association down to rs897876 on the predicted gene FLJ12164 on chromosome 2p14. SNP markers on genes which related to cardiovascular modulation near this region were analyzed. We found one SNP: rs11466212 in intron 5 of TGFA was highly correlated with rs897876 (r^2^ = 0.83) as well as pulse pressure (PP) (p-value = 0.007). Further studies will be required to clarify functional relation between FLJ12164 and TGFA responsible for blood pressure regulation. Interestingly, a recent linkage study investigating heritability of PP among Chinese twin pairs found3 linkage peaks on chromosomes 11, 12 and 18 [33], which are different from ours. However, instead of ambulatory BP monitoring, that study was based on a single-point BP value. Further studies with larger sample sizes using continuous BP recordings are needed to confirm our result.

In our study, the T allele of rs897876 located on 2p14 was independently associated with an increased risk of CVD in a prospective cohort. Currently, accumulating evidence demonstrates that ambulatory BP is a better predictor of morbidity and mortality than conventional BP [34]. Specifically, nighttime BP is more associated with an increased risk of cardiovascular events than daytime BP [35]. Nighttime BP, which has higher heritability, which is an indicator of a higher genetic component, is considered to have better predictive ability in determining clinical outcomes than daytime BP. Our study provided the first evidence linking genetic association with the clinical predictive value of ambulatory BP in clinical practice. Although only 204 (6.1%) young-onset hypertensive patients were included in the CVDFACTS cohort, the T allele of rs897876 was still associated with an increased risk of developing CAD and total cardiovascular events in the cohort, suggesting that the T allele of rs897876 could be a genetic prognostic factor for long-term outcomes in general cohorts that include hypertensive subjects. In our current study, we demonstrated the independent predictive value of rs897876 genotypes at chromosome 2p14 in determining future CV events. Like the famous loci in 9p21, which were identified in a GWAS that was not hypothesis-driven [36], [37] and was independent of traditional risk factors or family history [38], [39], the pathophysiological mechanism of rs897876 is not yet understood. Whether rs897876 contributes to CV risk through increasing PP or atherosclerosis or imparts a direct genetic effect on vascular damage needs to be clarified. It also remains to be determined whether nearby genes such as ACTR2, SPERD2 and RAB1A are responsible for the pathogenesis of CAD or if other mechanisms are involved.

There are some limitations to our study. There were not many significant associations between individual risk alleles and clinical events in the cohort. This result was expected given that alleles of small effect were tested in a community-based sample of modest size. Although none of the SNP markers achieved genome-wide significance in our first stage GWAS due to small sample size, the second stage replication study still revealed significant loci related to nighttime PP. Further studies with larger sample size and different ancestry groups followed by additional functional confirmation studies are warranted. The second limitation of this study is that ambulatory BP monitoring was not available in the CVDFACTS cohort. The finding that the T allele of rs897876 is associated with nighttime PP was found using ABP monitoring, but the association between 24-hour ambulatory BP parameters among subjects in CVDFACTs could not be evaluated.

In conclusion, we first identified rs897876 at 2p14 to be highly associated with nighttime PP in young-onset hypertension patients. Subjects carrying the TT genotype of rs897876 had a higher nighttime PP, suggesting that the T allele of rs897876 was an independent predictor in determining ambulatory nighttime PP. Although the function of this locus is not well understood, the CVDFACTS prospective cohort study clearly demonstrated that the TT genotype at rs897876 on 2p14 is significantly associated with an increased risk of future cardiovascular events. This suggests that rs897876 could be a genetic prognostic factor for cardiovascular events in a general cohort in Taiwan and that 2p14 may have an important role in the pathogenesis of cardiovascular diseases.

## Supporting Information

Figure S1 Multidimensional scaling (MDS) analysis plot. The MDS plot shows the first two principal components, estimated by PLINK, based on genotype data from 509,174 SNPs. No population stratification for YOH cases in the first stage was detected (Identify-by-state group-difference empirical p value = 0.99715 for T4: Case/case more similar).
(TIF)

Table S1 Top ten Eigen vectors of the covariance matrix between the initial stage and the second stage.
(DOCX)

Table S2 Baseline characteristics of patients with essential hypertension.
(DOCX)
