# Supplementary figures and images for: A Novel SNP Associated with Nighttime Pulse Pressure in Young-Onset Hypertension Patients Could Be a Genetic Prognostic Factor for Cardiovascular Events in a General Cohort in Taiwan
Source: PLoS One. 2014 Jun 3;9(6):e97919. doi: 10.1371/journal.pone.0097919 (PMC4043733; doi:10.1371/journal.pone.0097919)

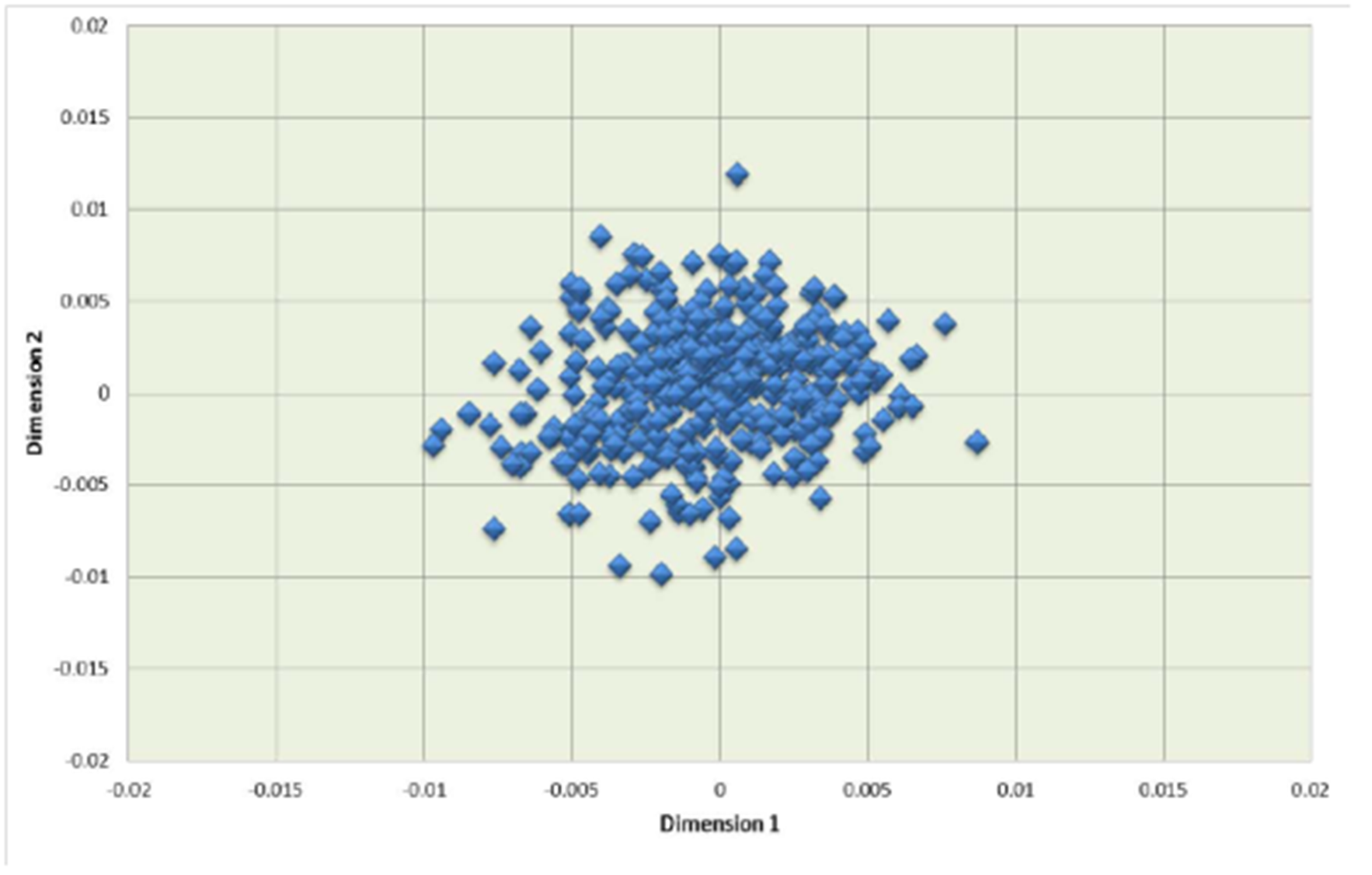

Supplement: Figure S1 — Multidimensional scaling (MDS) analysis plot. The MDS plot shows the first two principal components, estimated by PLINK, based on genotype data from 509,174 SNPs. No population stratification for YOH cases in the first stage was detected (Identify-by-state group-difference empirical p value = 0.99715 for T4: Case/case more similar). (TIF) [file pone.0097919.s001.tif]
