# Supplementary material for: A Novel SNP Associated with Nighttime Pulse Pressure in Young-Onset Hypertension Patients Could Be a Genetic Prognostic Factor for Cardiovascular Events in a General Cohort in Taiwan
Source: PLoS One. 2014 Jun 3;9(6):e97919. doi: 10.1371/journal.pone.0097919 (PMC4043733; doi:10.1371/journal.pone.0097919)
Supplement: Table S1 — Top ten Eigen vectors of the covariance matrix between the initial stage and the second stage. (DOCX) [file pone.0097919.s002.docx]

Table S1. the top ten Eigen vectors of the covariance matrix between the initial stage and the second stage

| **Eigenvector** | **P-value** |
| --- | --- |
| ev1 | 0.435238456 |
| ev2 | 0.522518236 |
| ev3 | 0.619521629 |
| ev4 | 0.177748442 |
| ev5 | 0.177201895 |
| ev6 | 0.435238456 |
| ev7 | 0.435238456 |
| ev8 | 0.8314846 |
| ev9 | 0.05546941 |
| ev10 | 0.435238456 |
