# Supplementary material for: A Novel SNP Associated with Nighttime Pulse Pressure in Young-Onset Hypertension Patients Could Be a Genetic Prognostic Factor for Cardiovascular Events in a General Cohort in Taiwan
Source: PLoS One. 2014 Jun 3;9(6):e97919. doi: 10.1371/journal.pone.0097919 (PMC4043733; doi:10.1371/journal.pone.0097919)
Supplement: Table S2 — Baseline characteristics of patients with essential hypertension. (DOCX) [file pone.0097919.s003.docx]

Baseline characteristics of patients with essential hypertension

|  | Hypertension onset  Before age 50 | Hypertension onset after age 50 |  |
| --- | --- | --- | --- |
|  | N=204 | N=185 | *P value* |
| Male, n (%) | 104 (50.9) | 97 (52.4) | 0.45 |
| BMI, kg/m^2^ | 26.1 ± 3.4 | 25.5 ± 2.9 | 0.002 |
| Diabetes, n (%) | 15 (7.4) | 20 (10.8) | 0.069 |
| Smoking, n (%) | 32 (15.7) | 33 (17.8) | 0.312 |
| Waist circumference, cm | 84.5 ± 9.1 | 85.8 ± 8.5 | 0.481 |
| SBP, mmHg | 138.2 ± 18.4 | 143.0 ± 20.2 | 0.160 |
| DBP, mmHg | 87.2 ± 11.4 | 80.9 ± 11.4 | 0.010 |
| Glucose, mg/dl | 105.7 ± 23.3 | 113.4 ± 38.0 | 0.000 |
| Triglycerides, mg/Dl | 132.0 ± 88.7 | 141.1 ± 129.5 | 0.031 |
| Cholesterol, mg/dL | 207.5 ± 41.9 | 211.4 ± 43.7 | 0.419 |
| HDL-C, mg/dL | 40.4 ± 10.8 | 40.5 ± 11.8 | 0.415 |
| LDL-C, mg/dL | 131.3 ± 36.2 | 133.5 ± 38.7 | 0.896 |
